# Edaravone Improves Septic Cardiac Function by Inducing an HIF-1*α*/HO-1 Pathway

**DOI:** 10.1155/2018/5216383

**Published:** 2018-03-22

**Authors:** Chao He, Wei Zhang, Suobei Li, Wei Ruan, Junmei Xu, Feng Xiao

**Affiliations:** ^1^Department of General Surgery, The Second Xiangya Hospital, Central South University, Changsha, Hunan 410011, China; ^2^Department of Anesthesiology, The Second Xiangya Hospital, Central South University, Changsha, Hunan 410011, China

## Abstract

Septic myocardial dysfunction remains prevalent and raises mortality rate in patients with sepsis. During sepsis, tissues undergo tremendous oxidative stress which contributes critically to organ dysfunction. Edaravone, a potent radical scavenger, has been proved beneficial in ischemic injuries involving hypoxia-inducible factor- (HIF-) 1, a key regulator of a prominent antioxidative protein heme oxygenase- (HO-) 1. However, its effect in septic myocardial dysfunction remains unclarified. We hypothesized that edaravone may prevent septic myocardial dysfunction by inducing the HIF-1/HO-1 pathway. Rats were subjected to cecal ligation and puncture (CLP) with or without edaravone infusion at three doses (50, 100, or 200 mg/kg, resp.) before CLP and intraperitoneal injection of the HIF-1*α* antagonist, ME (15 mg/kg), after CLP. After CLP, rats had cardiac dysfunction, which was associated with deformed myocardium, augmented lipid peroxidation, and increased myocardial apoptosis and inflammation, along with decreased activities of catalase, HIF-1*α*, and HO-1 in the myocardium. Edaravone pretreatment dose-dependently reversed the changes, of which high dose most effectively improved cardiac function and survival rate of septic rats. However, inhibition of HIF-1*α* by ME demolished the beneficial effects of edaravone at high dose, reducing the survival rate of the septic rats without treatments. Taken together, edaravone, by inducing the HIF-1*α*/HO-1 pathway, suppressed oxidative stress and protected the heart against septic myocardial injury and dysfunction.

## 1. Introduction

Sepsis, a systemic deleterious inflammatory response to infection or injury [1], has long been associated with high mortality rate, which mainly results from multiorgan dysfunction and failure [2]. Of note, cardiac dysfunction is highly prevalent during sepsis, which is a major cause of high mortality rate in septic patients [3–6]. However, therapy for this lethal disease is nonspecific and often not effective as current understanding of its pathophysiology remains elusive [7].

Existing evidence indicates that several mechanisms are often associated with septic myocardial dysfunction, including exaggerated oxidative stress, cardiomyocyte apoptosis, contractile dysfunction of the heart, and persistent inflammation [8, 9]. Among these, oxidative stress has been considered a critical contributor in promoting the progression of sepsis-induced organ failure, including myocardial dysfunction [10–12]. Therefore, therapies that can reduce oxidative stress may effectively attenuate the development and progression of septic myocardial dysfunction. Edaravone (EDA), a potent radical scavenger, is clinically employed in stroke patients; moreover, it has been identified as a potential protective agent for cardiovascular diseases result from inflammation, oxidative stress and/or cytokine-induced apoptosis, such as ischemic or diabetic cardiomyopathy [13, 14]. In sepsis, while the antioxidative effect of EDA has been documented in brain [15], pulmonary [16], liver [17], and renal injuries [18], less is known of its effect in septic cardiac complications.

It has been suggested that EDA exerts cardioprotective effects in ischemia/reperfusion injury by scavenging reactive oxygen species (ROS), which then reduces cardiac lipid peroxidation and cardiomyocyte apoptosis [14]. One of the major pathways regulated by ROS relies on the hypoxia-inducible transcription factor-1 (HIF-1), which regulates a myriad of genes that control cellular processes essential to the cardiovascular system, including metabolism, angiogenesis, cell survival, and oxygen delivery [19]. Activation of HIF-1*α*, the major functional subunit of HIF-1, has been found cardioprotective by inducing heme oxygenase (HO)-1 [20]. Indeed, HIF-1 is a key regulator of HO-1 which has been identified as one of the most important cardioprotective proteins in a wide variety of tissues in response to oxidative stress and inflammation [20, 21]. Of note, although evidence associating EDA and HIF-1*α* in cardiac diseases has not been documented in current literature, EDA has been found to regulate HIF-1*α* in neurological studies related to stroke [15]. Hence, we postulate that EDA may alleviate septic myocardial dysfunction by regulating the HIF-1/HO-1 pathway.

## 2. Materials and Methods

### 2.1. Animals

Sprague–Dawley rats (280–320 g) were obtained from the animal center of the Second Xiangya Hospital of Central South University. Rats were housed in individual cages with alternating 12-hour light/dark cycles in a temperature-controlled specific pathogen-free (SPF) environmental room, in which rats were acclimated for one week before experiment. Animals were fasted for 8 hours but had free access to water before the experiments. All animal care and experimental protocols complied with the Guidelines of Central South University for Animal Experimentation and were approved by the Institutional Animal Care and Use Committee at Central South University. All experimental procedures complied with the Guide for the Care and Use of Laboratory Animals (1996).

### 2.2. Experimental Protocol

The current experiment was conducted in two parts. In the first part, rats were allocated to five groups (*n* = 8 per group): sham (Sham), cecal ligation and puncture (CLP), and low (CLP + L-EDA), medium (CLP + M-EDA), or high dose (CLP + H-EDA) of the edaravone group. Rats were infused slowly with saline (2 mL) or 50 (low), 100 (medium), 200 (high) mg/kg edaravone [22] 10 minutes before CLP. In the second part, rats were allocated to four groups (*n* = 8 per group): Sham, CLP, CLP + H-EDA, and CLP + H-EDA+ 2-methoxyestradiol (ME). ME was injected intraperitoneally at 15 mg/kg (Sigma Aldrich, St. Louis, MO, USA) immediately after CLP in the treatment group. In the sham group, animals have no CLP but have vehicle treatment.

Cardiac function was evaluated by echocardiography under inhaled anesthesia with 1–3% isoflurane and 40% oxygen at 18 hours after operation. Briefly, a heparin saline-filled catheter (500 U/mL) was inserted from the right carotid artery into the left ventricle. Left ventricular systolic pressure (LVSP), left ventricular end diastolic pressure (LVEDP), maximal rate of the increase of left ventricular pressure (+dp/dt_max_), and maximal rate of the decrease of left ventricular pressure (−dp/dt_max_) were recorded by using a PowerLab (4S, Australia) as described previously [23].

### 2.3. Evaluation for Survival Rate

A cohort of rats (*n* = 16 per group) receiving the same protocols as in the part two of the experiment were used for survival rate evaluation. Rats in each group had free access to food and water and were kept under pathogen-free conditions. A video recorder was used to monitor the animals, and the survival rate was evaluated for 72 hours.

### 2.4. Cecal Ligation and Puncture (CLP)

CLP was performed as described previously [23]. Rats under general anesthesia underwent an abdominal midline incision after disinfection of the abdomen with 10% povidone iodine. The cecum was exposed and subjected to ligation just below the ileocecal valve to maintain bowel continuity. After puncturing twice with an 18-gauge needle, a small amount of bowel content was expelled from the punctures by manual application of sufficient pressure. Then the cecum was returned to the peritoneal cavity, and the abdominal incision was closed in 2 layers. At the end of surgery, 5 mL/kg of 0.9% NaCl was subcutaneously injected in the back. For the sham-operated animals serving as controls, the cecum was mobilized but no ligation or puncture was performed.

### 2.5. Histopathology Analysis

Rats were sacrificed at 18 hours after surgery by carbon dioxide inhalation. The left ventricular myocardial tissues were collected. Tissue sections of the myocardium were stained with hematoxylin-eosin (H&E) stain, and histological changes were evaluated by microscopy at 400x magnification.

### 2.6. Immunohistochemistry Assay

After deparaffination and microwave antigen reparation, sections were pretreated with 3% H_2_O_2_ for 20 minutes to reduce the endogenous peroxidase activity. Then the sections were ordinally incubated with the blocking buffer (10% normal goat serum) at room temperature for 1 hour, then with primary antibodies of 4-hydroxynonenal (HNE) (1/100 dilution; Abcam, Cambridge, UK) at 4°C overnight, followed with biotinylated secondary antibody (1 : 200, Santa Cruz Biotechnology, Inc., Santa Cruz, CA, USA) and avidine-biotinylated peroxidase complex (Vectostain ABC-Kit, Vector Lab, Burlingame, CA, USA) at room temperature for 1 hour. Coloration of sections was processed with diaminobenzidine (DAB, Vector Laboratories, Burlingame, CA, USA) and finished with distilled water. After counterstaining with hematoxylin for 30 seconds, sections were dehydrated with graded ethanol, cleared with dimethylbenzene, and mounted with neutral gums. Figures were captured with a Ti-S inverted microscope (Nikon, Japan) and analyzed with Image-Pro Plus software (version 6.0, Media Cybernetics, USA).

### 2.7. TUNEL for DNA Fragmentation

Terminal deoxynucleotidyl transferase dUTP nick-end labeling (TUNEL) staining was performed according to the manufacturer's protocol of DeadEnd™ Fluorometric TUNEL System kit (Promega, Madison, WI, USA). Sections were incubated with proteinase K solution (20 *μ*g/mL in PBS) for 10 minutes after deparaffination. TUNEL labeling was conducted with a mix of a 45 *μ*L equilibration buffer, 5 *μ*L nucleotide mix, and 1 *μ*L recombinant terminal deoxynucleotidyl transferase (rTdT) enzyme in a humidified, lucifugal chamber for 1 hour at 37°C. The slides were protected from direct light from this step to the end of experiment. Hoechst 33258 (H-33258, Sigma-Aldrich; 5 mg/mL in distilled water, 3 minutes) was used to stain nuclei and terminate the TUNEL reaction. Antifade solution was dropped to the area which was mounted by glass coverslips with clear nail polish sealing the edges. The samples were immediately analyzed under a fluorescence microscope using a standard fluorescein filter set to view the green fluorescence of fluorescein at 520 nm and blue Hoechst at 460 nm, then stored at 4°C in dark if necessary.

### 2.8. Thiobarbituric Acid Reactive Substance (TBARS) Assay for MDA

The MDA concentration was tested by TBARS Parameter™ Kit for measuring oxidative stress (R&D Systems, Minneapolis, USA). All of the steps were strictly performed according to the manufacturer's instruction. In brief, acid-treated samples and standards were added to the included 96-well microplate followed by the TBA reagent. Then the 96-well microplate was incubated at 45°C for 2 hours. The microplate was read at 532 nm, and the intensity of the color corresponds to the level of lipid peroxidation in the sample.

### 2.9. ELISA for Catalase, HO-1, and HIF-1*α* Activity

The activity of catalase, HO-1, and HIF-1*α* was measured in myocardial tissue lysates using colorimetric sandwich ELISA according to the vendor's instructions. All ELISA kits were purchased from LifeSpan BioSciences, Inc., USA.

### 2.10. Cytokine Measurement by ELISA

Heart tissues were homogenized and then centrifuged. The levels of IL-1*β*, IL-6, and TNF-*α* in heart homogenates were measured using ELISA kits according to the manufacturer's instructions (LifeSpan BioSciences, Inc., USA). The concentrations of the cytokines were quantified by referring to standard curves.

### 2.11. Western Blot

For nuclear purification, nuclear-cytosol extraction kit (Applygen Technologies, Beijing, China) was used according to the manufacturer's instruction. Briefly, the mixture was added with the cytosol extraction buffer supplemented with protease inhibitor cocktail and incubated on ice for 10 minutes. The mixture was then centrifuged at 1000*g* for 5 minutes and the resulted pellet contained crude nuclei. The cytosol fraction was extracted by further centrifuging the supernatant at 12000*g* for 5 minutes. The crude nuclear pellet was incubated in the nuclear extraction buffer on ice for 30 minutes, with vortexing for 10 seconds every 5 minutes, then centrifuged at 12000*g* for 5 minutes. The resulting supernatant contained the nuclear fraction.

Samples were homogenized, and protein concentrations were determined using the BCA protein assay (Bio-Rad, Hemel Hempstead, Hertfordshire, UK). Fifty micrograms of each prepared sample was separated by 8 or 12% sodium dodecylsulfate polyacrylamide gel electrophoresis, then transferred to polyvinylidene fluoride membranes (EMD Millipore Corporation, Billerica, MA, USA), and incubated with a blocking solution composed of 5% bull serum albumin (BSA) in Tris-buffered saline with Tween (pH 8.0, 10 mm Tris, 150 mm NaCl, 0.1% Tween, TBST). Membranes were incubated overnight (14–16 hours) at 4°C with the corresponding primary antibodies: anti-HIF-*α* (1 : 2000), anti-Lamin B2 (1 : 1000), anti-HO-1 (1 : 2000), and anti-GAPDH (1 : 1000). All antibodies were purchased from Cell Signaling Technology, Beverly, MA, USA. After being washed with TBST, samples were incubated with the corresponding secondary antibodies for 1 hour at room temperature. Immunoreactive proteins were visualized using the ECL Western blotting system (Pierce Biotechnology, Rockford, IL, USA) and scanned by an Image Master II scanner (GE Healthcare, Milwaukee, WI, USA). All the images were analyzed using ImageQuant™ TL software v2003.03 (GE Healthcare, Milwaukee, WI, USA).

### 2.12. Statistical Analysis

Data were presented as mean ± standard deviation (SD) and processed by GraphPad Prism 6.0 software. One-way analysis variance (ANOVA) followed by post hoc analysis (Tukey test) was used for comparison between groups after confirmation of homoscedasticity. *P* value less than 0.05 (*P* < 0.05) was considered statistically significant.

## 3. Results

### 3.1. EDA Prevents Sepsis-Induced Myocardial Injury and Cardiac Dysfunction

**Figure 1:**
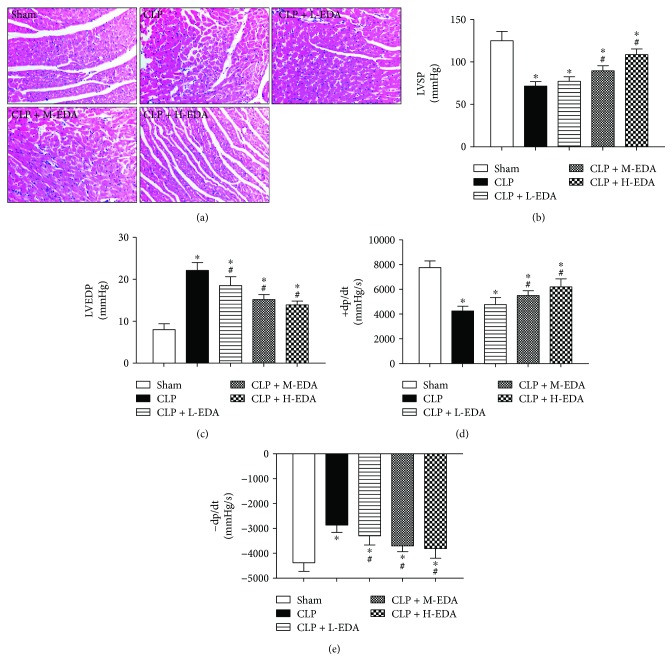
H&E staining of the myocardium and indices of cardiac function in septic rats. Edaravone (EDA) at low (L), medium (M), or high (H) dose was injected intravenously 10 minutes before CLP, respectively. Myocardium architecture was visualized by (a) H&E staining. Cardiac function was demonstrated in terms of (b) left ventricular systolic pressure (LVSP), (c) left ventricular end diastolic pressure (LVEDP), (d) maximal slope of left ventricular systolic pressure increment (+dP/dt_max_), and (e) maximal slope of left ventricular diastolic pressure decrement (−dP/dt_max_). ^∗^*P* < 0.05 versus Sham. ^#^*P* < 0.05 versus CLP. Data are presented as mean ± SEM. *n* = 8 per group.

To assess whether EDA prevents sepsis-induced cardiac injury, EDA was infused at low, medium, or high doses (50, 100, or 200 mg/kg, resp.) 10 minutes prior to CLP surgery. As demonstrated in the H&E staining of the myocardium (Figure 1(a)), the normal architecture of the myocardium as shown in the sham group was deformed after CLP. Pretreatment with high dose of EDA effectively preserved the normal conformation of the myocardium, while low and medium doses of EDA did not. Functional-wise, CLP-induced sepsis severely compromised cardiac function, evidenced by reduced LVSP, dP/dt_max_, −dP/dt_max_ (Figures 1(b), 1(d), and 1(e)), and increased LVEDP (Figure 1(c)) (CLP versus Sham, *P* < 0.05). Administration of all three doses of EDA improved cardiac function with effectiveness ascending from low to high doses of EDA. EDA at high dosage was found to be the most effective among the tested dosages in prevention of cardiac dysfunction induced by CLP.

### 3.2. EDA Reduces Myocardial Oxidative Stress in Septic Rats

**Figure 2:**
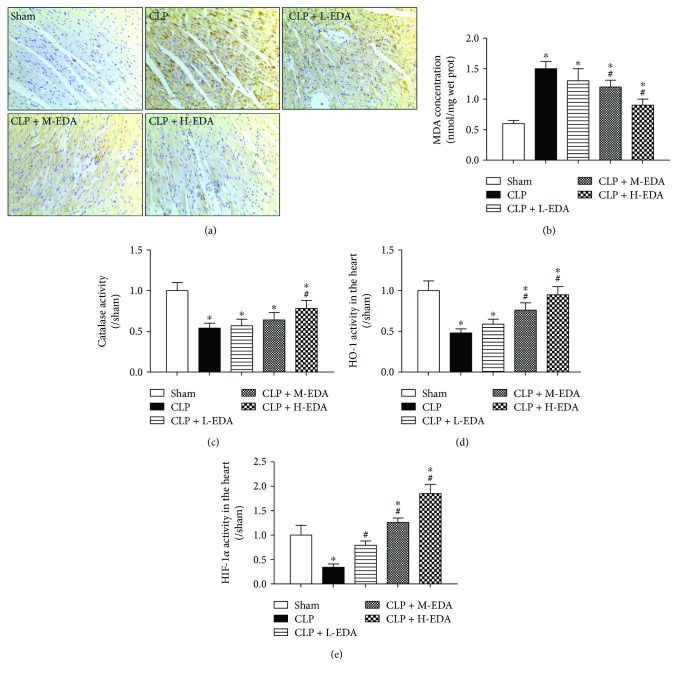
4-HNE staining of the myocardium and the cardiac level of oxidative stress markers in septic rats. Edaravone (EDA) at low (L), medium (M), or high (H) dose was injected intravenously 10 minutes before CLP, respectively. Lipid peroxidation level in the myocardium was visualized by (a) 4-HNE staining. Cardiac oxidative stress was indicated by levels of (b) MDA concentration, (c) catalase activity, (d) HO-1 activity, and (e) HIF-1*α* activity in the myocardium. ^∗^*P* < 0.05 versus Sham. ^#^*P* < 0.05 versus CLP. Data are presented as mean ± SEM. *n* = 8 per group.

Sepsis damages myocardium integrity which is associated with upsurge of oxidative stress [24]. After CLP, increased lipid peroxidation was found in the heart as demonstrated in the 4-HNE staining of the myocardium in Figure 2(a), which was accompanied by a significantly increased MDA level (Figure 2(b)) and decreased catalase, HIF-1*α*, and HO-1 activities (Figures 2(c)–2(e)) (CLP versus Sham, *P* < 0.05). Pretreatment of EDA minimized these changes in a dose-dependent manner that EDA at high dosage most effectively reduced lipid peroxidation and the MDA level and enhanced activities of catalase, HIF-1*α*, and HO-1 (CLP + H-EDA versus CLP, *P* < 0.05).

### 3.3. Inhibition of HIF-1*α* Represses the Antioxidative Effect of EDA in Septic Rats

**Figure 3:**
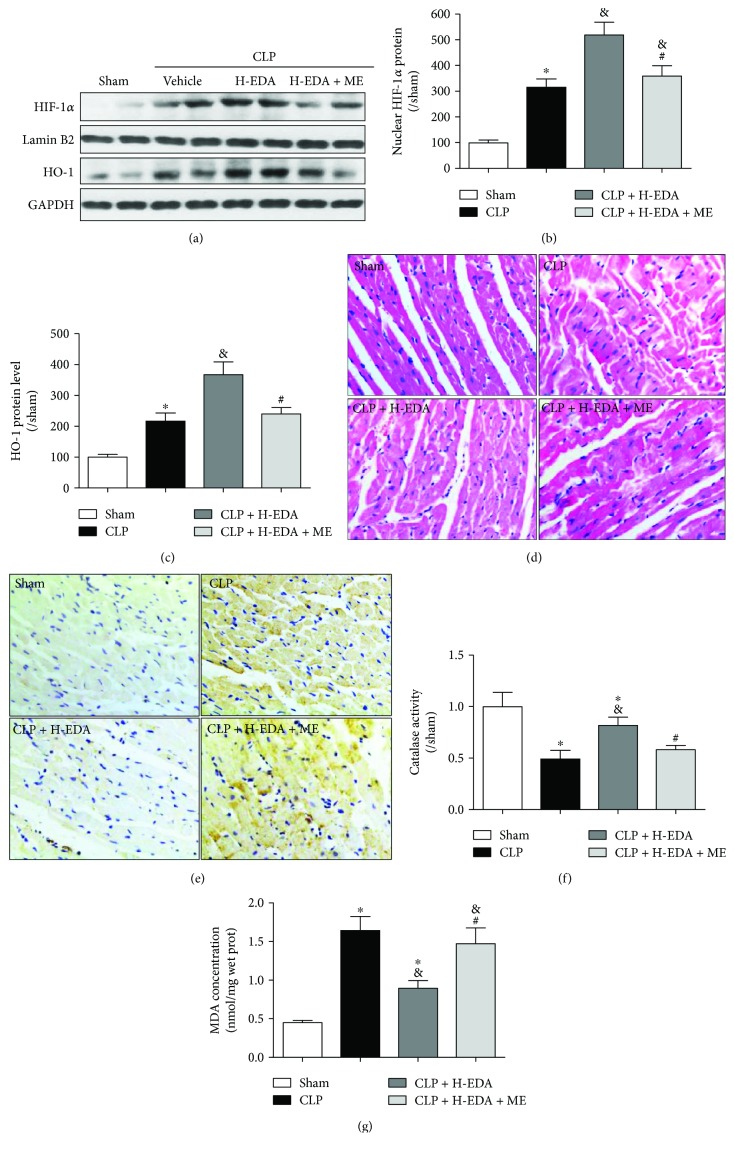
Effect of the HIF-1*α* antagonist on oxidative stress in edaravone-pretreated septic rats. Edaravone (EDA) at high (H) dose was injected intravenously 10 minutes before CLP, while the HIF-1*α* antagonist, ME, was injected intraperitoneally after CLP. Protein expressions of (a–c) HIF-1*α* and HO-1 were assessed in the myocardium. Myocardium architecture and lipid peroxidation level were visualized by (d) H&E staining (×400) and (e) 4-HNE staining (×200), respectively. Levels of (f) MDA concentration and (g) catalase activity were also evaluated in the myocardium. ^∗^*P* < 0.05 versus Sham. ^&^*P* < 0.05 versus CLP. ^#^*P* < 0.05 versus CLP + H-EDA.

EDA has been suggested to modify HIF-1*α* binding activity in astrocyte [25]; however, whether EDA acts via HIF-1*α* in cardiac injury remains unknown. In CLP-induced sepsis, we observed a significant decrease in protein expressions of HIF-1*α* in the nucleus along with HO-1 in the heart (CLP versus Sham, *P* < 0.05) (Figures 3(a)–3(c)). Pretreatment with H-EDA not only prevented the suppression of HIF-1*α* and HO-1 but also enhanced their expressions, which reduced the anatomical distortion (H&E stain) and lipid peroxidation (4-HNE stain) in the myocardium after CLP (Figures 3(d) and 3(e)). However, the administration of the HIF-1*α* inhibitor, ME, abolished these effects of H-EDA in the myocardium (H-EDA + ME versus H-EDA, *P* < 0.05). Deteriorated architecture of the myocardium and increased lipid peroxidation were observed following the inhibition of HIF-1*α*. Further, besides HO-1, catalase activity and MDA concentration in the heart were significantly lower in the CLP + H-EDA + ME group compared to the CLP + H-EDA group (H-EDA + ME versus H-EDA, *P* < 0.05), suggesting that the antioxidative effect of EDA was reversed by the HIF-1*α* inhibitor (Figures 3(f) and 3(g)). The presented data implicates that HIF-1*α* inhibition can reverse the cardioprotective effect of EDA even at high dosage.

### 3.4. Inhibition of HIF-1*α* Diminishes the Antiapoptotic Effect of EDA and Aggravated Cardiac Dysfunction in Septic Rats

**Figure 4:**
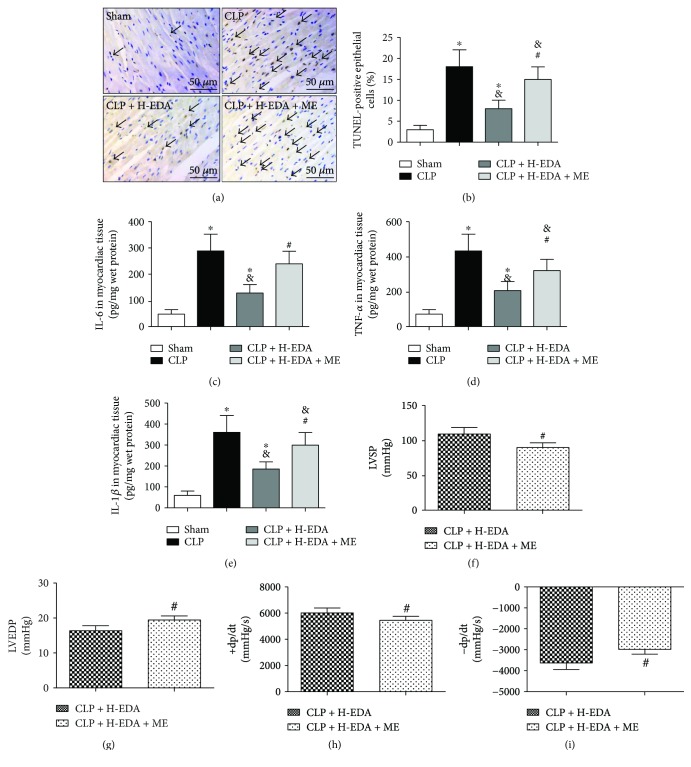
Effect of the HIF-1*α* antagonist on cardiomyocyte apoptosis and cardiac function in edaravone-pretreated septic rats. Edaravone (EDA) at high (H) dose was injected intravenously 10 minutes before CLP, while the HIF-1*α* antagonist, ME, was injected intraperitoneally after CLP. Cardiomyocyte apoptosis was assessed by terminal deoxynucleotidyl transferase dUTP nick-end labeling (TUNEL). (a and b) TUNEL-positive cells were stained brown and counted. Black arrows means TUNEL positive cell. Cytokines IL-6 (c), TNF-*α* (d), and IL-1*β* (e) were detected by an ELISA method. ^∗^*P* < 0.05 versus Sham. ^&^*P* < 0.05 versus CLP. ^#^*P* < 0.05 versus CLP + H-EDA. Cardiac function was demonstrated in terms of (f) left ventricular systolic pressure (LVSP), (g) left ventricular end diastolic pressure (LVEDP), (h) maximal slope of left ventricular systolic pressure increment (+dP/dt_max_), and (i) maximal slope of left ventricular diastolic pressure decrement (−dP/dt_max_). ^#^*P* < 0.05 versus CLP + H-EDA. Data are presented as mean ± SEM. *n* = 8 per group.

Cardiomyocyte apoptosis has been closely linked to sepsis-induced myocardial injury, as well as deleterious inflammatory response in the heart, which leads to heart failure [26]. As demonstrated in the TUNEL assay in the myocardium (Figure 4(a)), TUNEL-positive cells were stained brown (labeled by arrows) as we observed significantly more apoptotic cells in the CLP group compared to the sham group (CLP versus Sham, *P* < 0.05) (Figure 4(b)). Pretreatment of EDA at high dosage markedly reduced the number of apoptotic cardiomyocytes (CLP + H-EDA versus CLP, *P* < 0.05). However, the administration of ME to EDA-pretreated septic rats diminished the antiapoptotic effect of EDA as the number of apoptotic cardiomyocytes was significantly higher in the CLP + H-EDA + ME group than in the CLP + H-EDA group (CLP + H-EDA versus CLP + H-EDA + ME, *P* < 0.05). Additional measurement of the inflammatory markers, IL-6, TNF-*α*, and IL-1*β*, in the myocardium revealed upsurge of their expressions after CLP (CLP versus Sham, *P* < 0.05) (Figures 4(c)–4(e)). While H-EDA pretreatment significantly reduced the inflammatory markers (CLP + H-EDA versus CLP, *P* < 0.05), injection of the HIF-1*α* inhibitor reversed the anti-inflammatory effect of H-EDA (CLP + H-EDA versus CLP + H-EDA + ME, *P* < 0.05).

EDA-improved cardiac function was also demoted by the HIF-1*α* inhibitor. As demonstrated in Figures 4(g)–4(i), the inhibition of HIF-1*α* by ME in H-EDA-pretreated septic rats significantly reduced LVSP, +dp/dt, and −dp/dt and increased LVEDP with respect to the high-dose EDA-treated group (CLP + H-EDA + ME versus CLP + H-EDA, *P* < 0.05).

### 3.5. HIF-1*α* Inhibition Abolished the Survival-Promoting Effect of EDA in Septic Rats

**Figure 5:**
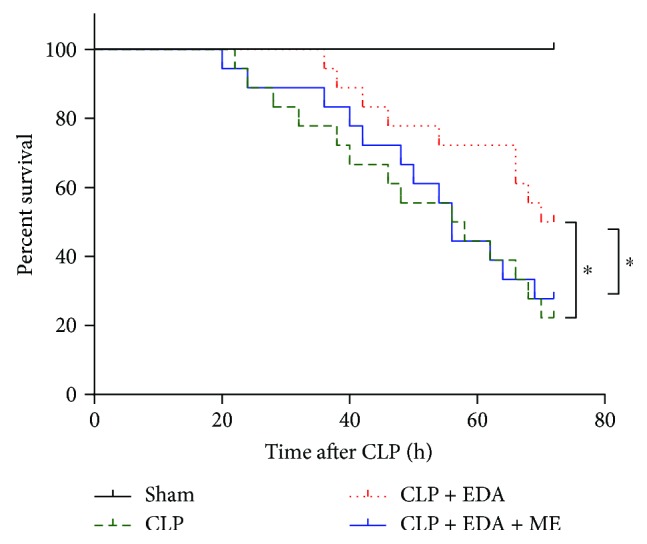
Survival rates of rats after CLP. Rats were subjected to CLP with or without pretreatment of high (H) dose of edaravone (EDA) and posttreatment of the HIF-1*α* antagonist, ME. Survival rate were monitored within 72 hours after CLP. ^∗^*P* < 0.05 versus CLP + H-EDA. Data are presented as mean ± SEM. *n* = 16 per group.

Performance of cardiac function is a key predictor of survival in sepsis [27]. As we have found that EDA provided cardioprotection in septic rats, here we determined whether EDA pretreatment can improve the survival rate of septic rats after CLP. As shown in Figure 5, survival rate of rats dropped to 20% at 72 hours after CLP. Pretreatment of EDA significantly increased survival rate to nearly 50% (CLP + H-EDA versus CLP, *P* < 0.05), whereas the administration of the HIF-1*α* antagonist reversed the survival rate to as low as in the CLP group (CLP + H-EDA + ME versus CLP + H-EDA, *P* < 0.05).

## 4. Discussion

In this study, we have shown that EDA can alleviate septic myocardial dysfunction by reducing cardiac oxidative stress through the HIF-1*α*/HO-1 pathway. In the present CLP model of sepsis in the rat, we observed lethal damage in animal's myocardial morphology and function, manifested by destructed architecture of the myocardium, cardiomyocyte apoptosis, reduced LVSP, dP/dt_max_, and −dP/dt_max_, and increased LVEDP. These alterations paralleled alterations in oxidative stress that we found significantly augmented lipid peroxidation along with decreased activities of catalase, HIF-1*α*, and HO-1 in the heart. Intravenous injection of EDA before CLP reversed the alterations in a dose-dependent manner. Moreover, the beneficial effects of EDA, even at high dose, can be demolished by the HIF-1*α* inhibitor, ME. In particular, the inhibition of HIF-1*α* reverted the high-dose EDA-induced protein expressions of HIF-1*α* and HO-1 and the EDA-reduced cardiomyocyte apoptosis. Our findings suggest that EDA, by inducing the HIF-1*α*/HO-1 pathway in advance of sepsis, can reduce cardiac oxidative stress and prevent septic cardiac dysfunction, which eventually improves animal survival.

Sepsis is a systemic deleterious inflammatory response to infection or injury, in which lung dysfunction is the primary detrimental effect, while dysfunction of other organs such as the heart and kidneys is secondary to lung dysfunction, and also in other situations, cardiac dysfunctions (e.g., heart failure, arrhythmia, and myocardial ischemia) often happen secondary to an abrupt worsening of renal function [28] or lung function [7]. In these regards, exploration of effective means protecting the heart against cardiac dysfunction that can be applied before or after septic cardiac dysfunction is also an important topic and worth of investigation. The current study was aimed at testing the effect of edaravone for pretreatment before septic myocardial dysfunction, which may be more of worth in preventing septic myocardial dysfunction. And our results were consistent with those of the studies of Chen et al. [29] and Chen et al. [30], which showed that trimetazidine and baicalein pretreatment protected the heart from septic myocardial dysfunction.

Our observations extend beyond the current literature on EDA, which has mainly documented its pleiotropic therapeutic effect in ischemia/reperfusion injury in various organs by scavenging ROS [14], among which, a few studies on brain ischemia/reperfusion injury have related EDA to HIF-1*α* signaling, which suggests that EDA posttreatment represses HIF-1*α* in the neuron [15, 31]. Here, for the first time, we demonstrated that EDA pretreatment induces HIF-1*α* in the myocardium and prevents myocardial dysfunction during sepsis. The discrepancy between our finding and the literature may due to differences in cell type and cellular microenvironment, which are thought to be crucial in the regulation of HIF-1*α* by ROS [32]. During oxidative stress, cellular treatment with ROS scavengers significantly inhibits HIF-1*α* protein expression and activity in a range of cell types, including myocardial cells [33]. On the other hand, during normoxia, the application of antioxidant either has no effect on HIF-1*α* or increases HIF-1*α* expression and activity [34, 35]. Therefore, because previous studies administered EDA to animals after ischemia/reperfusion injury when the microenvironments were under tremendous oxidative stress, HIF-1*α* was repressed as ROS was by EDA [15, 31].

In contrast, our study treated animals with EDA before CLP injury when the cardiomyocyte was under normoxic condition, thus HIF-1*α* was induced by EDA. As a matter of fact, earlier studies on the regulation of HIF-1 in response to cellular redox states showed that hydroxyl radicals mediate the inhibition of HIF-1*α* activity and possibly the degradation of HIF-1*α*, suggesting that HIF-1 DNA binding requires reducing conditions [35]. Since EDA has been reported to exert antioxidative effects by quenching hydroxyl radicals and hydroxyl radical-dependent lipid peroxidation [14], it is possible that, in our study, EDA pretreatment induced HIF-1*α* by scavenging hydroxyl radicals, which also explains the reduced lipid peroxidation in the myocardium of animals treated with EDA.

Indeed, the induction of HIF-1*α* has been found to be cardioprotective as HIF-1*α* is a key regulator of HO-1, one of the most important cardioprotective proteins in a panoply of tissues and conditions [20, 36]. HO-1 catalyzes heme oxidation and gives rise to CO, bilirubin, and ferritin, all of which contributes to cellular mechanisms against oxidative damage and death [21] that involve upregulation of catalase [37]. The previous study demonstrated that cardiac-specific overexpression of HO-1 alleviates myocardial ischemia-reperfusion injury [38]. Further, cardiac preconditioning with the HIF-1 activator attenuates postischemic myocardial injury [20]. Therefore, HO-1 is thought to provide both immediate and delayed protections against ischemia-reperfusion injury [36]. In our study, EDA pretreatment dose-dependently induced HIF-1*α*, HO-1, and catalase activities, which was associated with reduced lipid peroxidation, cardiomyocyte apoptosis, and MDA level in the myocardium together with improved cardiac function at 18 hours after CLP. The delayed protective effect is even prominent in the survival study where we observed about 30% higher survival rate with EDA pretreatment than that without treatment at 72 hours after CLP. These beneficial effects of EDA were attenuated by the HIF-1*α* inhibitor, suggesting that EDA preconditions the heart against septic cardiac dysfunction via upregulation of HIF-1*α* which in turn boosts HO-1 expression and exerts cardioprotective effects.

Of note, the mechanisms of the effect of edaravone have been reported to be mediated by enhancement of nitric oxide (NO) [39, 40], which may in turn result in a protective effect in injured cardiac tissues. On the other hand, under normal oxygen tension, the master transcriptional factor HIF-1 activity is usually suppressed due to the rapid, oxygen-dependent degradation of one of its two subunits, HIF-1*α*. Normoxic HIF-1 activity can be upregulated through NO-mediated S-nitrosylation and stabilization of HIF-1*α*. So, the EDA may increase NO generation to activate the HIF-1*α*/HO-1 pathway which takes part in the septic cardioprotective progress. And the *in vitro* study will be performed in the future study to verify our hypothesis. On the other hand, clinically, edaravone as an approved treatment for acute ischemic stroke (AIS) in Japan is recommended by the American Heart Association in the guidelines for the early management of AIS patients [41]. In sepsis, while the preventive/protective effect of edaravone has been demonstrated in the lung, liver, and kidney [16, 17, 42], we sought for its efficacy on cardiac complication, which is a leading cause of death in septic patients. We believe our study has strengthened the support of early application of edaravone in sepsis to reduce the occurrence of cardiac dysfunction. It is of notice that intravenous infusion of edaravone in patients with septic peritonitis for 2 weeks starting from their admission to intensive care unit has been shown to improve inflammatory and oxidative states with better patient outcomes [43]. Thus, it is possible that posttreatment with edaravone is clinically cardioprotective but needs further investigations.

In summary, we have demonstrated for the first time that pretreatment with EDA induces preconditioning-like protection effect in the heart against septic myocardial injury and dysfunction. By inducing the HIF-1*α*/HO-1 pathway, EDA primed the heart with an active cellular antioxidative mechanism which involves an increased catalase activity and a decreased MDA level in the myocardium. Given that EDA has been used clinically to treat ischemic stroke, the advancing knowledge we have provided here on the cardiac preconditioning effect of EDA via HIF-1*α*/HO-1 activation in sepsis may support a novel use of EDA as a treatment for septic patients.
